# A highly efficient pipeline for protein expression in *Leishmania tarentolae *using infrared fluorescence protein as marker

**DOI:** 10.1186/1475-2859-9-29

**Published:** 2010-05-10

**Authors:** Hakan Dortay, Bernd Mueller-Roeber

**Affiliations:** 1 University of Potsdam, Institute of Biochemistry and Biology, Karl-Liebknecht-Straße 24-25, Haus 20, 14476 Potsdam-Golm, Germany; 2 Max-Planck Institute of Molecular Plant Physiology, Am Mühlenberg 1, 14476 Potsdam-Golm, Germany

## Abstract

**Background:**

*Leishmania tarentolae*, a unicellular eukaryotic protozoan, has been established as a novel host for recombinant protein production in recent years. Current protocols for protein expression in *Leishmania *are, however, time consuming and require extensive lab work in order to identify well-expressing cell lines. Here we established an alternative protein expression work-flow that employs recently engineered infrared fluorescence protein (IFP) as a suitable and easy-to-handle reporter protein for recombinant protein expression in *Leishmania*. As model proteins we tested three proteins from the plant *Arabidopsis thaliana*, including a NAC and a type-B ARR transcription factor.

**Results:**

IFP and IFP fusion proteins were expressed in *Leishmania *and rapidly detected in cells by deconvolution microscopy and in culture by infrared imaging of 96-well microtiter plates using small cell culture volumes (2 μL - 100 μL). Motility, shape and growth of *Leishmania *cells were not impaired by intracellular accumulation of IFP. In-cell detection of IFP and IFP fusion proteins was straightforward already at the beginning of the expression pipeline and thus allowed early pre-selection of well-expressing *Leishmania *clones. Furthermore, IFP fusion proteins retained infrared fluorescence after electrophoresis in denaturing SDS-polyacrylamide gels, allowing direct in-gel detection without the need to disassemble cast protein gels. Thus, parameters for scaling up protein production and streamlining purification routes can be easily optimized when employing IFP as reporter.

**Conclusions:**

Using IFP as biosensor we devised a protocol for rapid and convenient protein expression in *Leishmania tarentolae*. Our expression pipeline is superior to previously established methods in that it significantly reduces the hands-on-time and work load required for identifying well-expressing clones, refining protein production parameters and establishing purification protocols. The facile in-cell and in-gel detection tools built on IFP make *Leishmania *amenable for high-throughput expression of proteins from plant and animal sources.

## Background

*Leishmania tarentolae *is a protozoon of the genus Trypanosoma, and a parasite of the gecko *Tarentolae annularis*. It has been established as a new eukaryotic expression system for recombinant protein production [1]. An interesting feature of proteins produced in *Leishmania *is their animal-like N-glycosylation pattern, as demonstrated for erythropoietin [1]. Systems for constitutive and regulated expression of heterologous proteins have been developed [1-3]. Compared to mammalian cell cultures, *Leishmania *has the advantage of a higher specific growth rate although cultivation in high cell densities (>2 × 10^8 ^cells/mL) with a high specific growth rate in serum-free medium was not possible initially. Recently, however, Fritsche *et al*. developed an alternative growth medium containing hemin, an iron-containing porphyrin essential for growth of *Leishmania tarentolae*, as the only animal ingredient [4]. Hemin has been shown to stimulate cell proliferation and protein synthesis in *L. donovani *[5].

Since its introduction as a new host for protein production which benefited from the development of methods for trypanosomatid cultivation and their genetic manipulation [6], *Leishmania tarentolae *has been used for the successful expression of various heterologous proteins such as e.g. proprotein convertase 4 (a member of Ca^2+^-dependent mammalian subtilases), human laminin-332 and a tissue type plasminogen activator [7-9]. Recently it was also shown that extracts generated from *Leishmania *cells can be used for protein expression *in vitro*; in ideal cases up to 300 μg/mL of recombinant protein could be produced within 2 h [10]. However, successful expression of plant proteins in *Leishmania *cells or *in vitro *extracts has to our knowledge not been reported so far.

Foldynová-Trantirková *et al*. have reported a protocol for cost-effective amino-acid-type-selective isotope labeling of proteins expressed in *Leishmania tarentolae *[11]. The method is based on cultivation of *Leishmania *cells in a relatively cheap complex medium supplemented with labeled amino acids. The procedure avoids expensive synthetic media.

Although *Leishmania *has been shown to be a suitable host for foreign protein expression, only a limited number of labs have so far established routine culture and expression pipelines for this organism. A major reason for this may be the lengthy procedure that is normally required to find good expressor clones. The presently suggested protocols for expression of recombinant proteins in *Leishmania *require a stepwise scale-up of the culture volume before a protein of interest can be detected among several randomly selected clones (for details see manual of the LEXSYcon2 Expression Kit offered by commercial supplier Jena Bioscience; http://www.jenabioscience.com). A typical scheme for setting up protein expression in *Leishmania *requires seven to eight days.

Recently, an infrared fluorescing protein (IFP) has been engineered as a new reporter protein, derived from a bacterial (*Deinococcus radiodurans*) phytochrome [12]. IFP covalently binds biliverdin, a natural product of heme catabolism involved in aerobic respiration, and becomes infrared fluorescent with excitation and emission maxima at 684 nm and 708 nm, respectively. Successful expression of IFP has been reported for *E. coli*, human embryonic kidney cells (HEK293A) and mice. As infrared light relatively well penetrates animal tissue, IFP is suitable for whole-body imaging with negligible background signal, as shown by visualization of liver-expressed IFP in intact mice [12].

Here we demonstrate that IFP can be employed as a suitable and ease-to-handle reporter protein in *Leishmania*. We developed a procedure that shortens the currently available protocol for protein expression in *Leishmania *and significantly reduces overall work load. The newly established work-flow qualifies for multi-parallel protein expression in *Leishmania *and thus has the potential to be employed in genomics and proteomics research for the functional analysis of proteins.

## Methods

### Chemicals

Biliverdin was purchased from Toronto Research Chemicals (North York, Ontario, Canada), and biliverdin hydrochloride was obtained from Frontier Scientific (Carnforth, Lancashire, UK). Hemin was ordered from Sigma-Aldrich (Deisenhofen, Germany).

### Constructs

The cDNAs encoding for IFP and the three *Arabidopsis thaliana *proteins ANAC42, ARR1 and TPK1 were amplified by PCR using, respectively, the pENTR1A-IFP1.4&GFP vector [12], *ANAC42 *cDNA [AGI code: AT2G43000], the pDONR201-ARR1 vector [13], or *TPK1 *cDNA [AGI: AT5G55630] as templates. ANAC42 and ARR1 are transcription factors, whereas TPK1 is an ion channel. For TPK1, a partial cDNA encoding the N-terminal part of the channel protein (amino acids 1-79) was used [14]. After amplification with PCR primers IFP sense, 5'-TCACCCATGGCTCGGGACCCTCTG-3' and IFP antisense, 5'-GTTGGTACCTTTATACAGCTCGTCCATTCC-3' IFP was cloned by restriction and ligation into the *Nco*I and *Kpn*I sites of the pLEXSY-sat2 vector (Jena Bioscience, Jena, Germany) encoding a C-terminal 6xHis epitope (fused to IFP) and a nourseothricin antibiotic resistance marker (Jena Bioscience). This vector, pLEXSY-IFP-His, was used to generate plasmids encoding C-terminally IFP-tagged ANAC42, ARR1 and TPK1 fusion proteins. To this end, *ANAC42*, *ARR1 *and *TPK1 *(1-79) sequences were PCR-amplified using the primers: ANAC42 sense, 5'-CACCCATGGGTGGCGAAGGTAACTTAGGTAAG-3', and ANAC42 antisense, 5'-TCACCCATGGCGGGTTTAGTGTTGCCATCTATAAC-3'; ARR1 sense, 5'-TCACCCATGGTGAATCCGAGTCACGGAAGAG-3', and ARR1 antisense, 5'-TCACCCATGGCAACCTGCTTAAGAAGTGCGCTC-3'; TPK1 sense, 5'-AAGAAGACATGTCGAGTGATGCAGCTC-3', and TPK1 antisense, 5'-AAGAAGACATGTCCACTCGCCTGAGATTCGG-3'. ANAC42- and ARR1-encoding PCR products were restricted and ligated into the *Nco*I site of vector pLEXSY-IFP-His. TPK1 (1-79)-encoding fragment was restricted by *Pci*I and ligated into the *Nco*I site of vector pLEXSY-IFP-His. The resulting constructs were named pLEXSY-ANAC42-IFP-His, pLEXSY-ARR1-IFP-His and pLEXSY-TPK1-IFP-His, respectively. The plasmids pLEXSY-IFP-ANAC42-His and pLEXSY-IFP-ARR1-His were generated in two steps for the expression of proteins N-terminally tagged with IFP. In the first step, ANAC42- and ARR1-encoding fragments were PCR amplified using the primers ANAC42 senseN, 5'-TCACCCATGGGTGGCGAAGGTAACTTAGGTAAG-3', and ANAC42 antisenseN 5'-TCCGCTAGCGGGTTTAGTGTTGCCATCTATAAC-3'; ARR1 senseN, 5'-TCACCCATGGTGAATCCGAGTCACGGAAGAG-3', and ARR1 antisenseN 5'-AAGAATGCTAGCAACCTGCTTAAGAAGTGCGCTC-3'. The resulting PCR products were then restricted and ligated into the *Nco*I and *Nhe*I sites of the pLEXSY-sat2 vector. These resulting constructs, pLEXSY-ANAC42-His and pLEXSY-ARR1-His, were then used in a second cloning step for the generation of the plasmids pLEXSY-IFP-ANAC42-His and pLEXSY-IFP-ARR1-His using the primers IFP sense and IFP antisense2, 5'-TCACCCATGGCTTTATACAGCTCGTCCATTCC-3', followed by restriction and ligation into the *Nco*I site of the vectors pLEXSY-ANAC42-His and pLEXSY-ARR1-His. pLEXSY-IFP-TPK1-His was generated using the primers TPK1 senseN, 5'-GTTGGTACCATGTCGAGTGATGCAGCTC-3' and TPK1 antisenseN, 5'-GTTGGTACCCACTCGCCTGAGATTCGG-3', followed by restriction and ligation into the *Kpn*I site of pLEXSY-IFP-His.

### Protein expression and purification

IFP-6xHis, IFP-ANAC42-/ARR1-/TPK1-6xHis and ANAC42-/ARR1-/TPK1-IFP-6xHis fusion proteins were expressed using the LEXSYcon2 Expression Kit (Jena Bioscience). If not explicitly pointed out all components for cultivation of *Leishmania *cells and protein expression are included in the kit. Plasmids were transfected into *Leishmania *cells by electroporation and transfected cells were selected on nourseothricin-supplemented selective plates after five to seven days of incubation at 26°C according to the protocol included in the expression kit. Individual clones were selected and transferred sequentially each second day after incubation at 26°C into 96-well microtiter plates, from there into 24-well deep-well plates and than into 25 cm^2 ^tissue culture flasks, filled with 150 μL, 1 mL and 10 mL selective medium. If not stated otherwise 96-well microtiter plates and 24-well deep-well plates were shaken at 60 rpm and tissue culture flasks were incubated in a static upright position. For protein stability and solubility tests 2 × 10 mL of an IFP expressing cell culture were pooled and centrifuged; cells were then sonicated in 1 mL standard Tris buffer in the absence or presence of protease inhibitors (1 mM EDTA, 1 mM PMSF, EDTA-free protease inhibitor cocktail; Roche, Mannheim, Germany). Supernatants of ultracentrifuged samples were analyzed. For protein purification, culture volume was scaled-up using nine 150 cm^2^-tissue culture flasks each containing 60 mL non-selective YE medium [4]. After pooling all cultures, cells were centrifuged and the cell pellet was either stored at -80°C until use or immediately resuspended in standard Tris buffer supplemented with protease inhibitors (1 mM PMSF, EDTA-free protease inhibitor cocktail). Resuspended cells were sonicated and the supernatant of centrifuged samples was used for purification. IFP-His protein was purified using a 1-mL HisTrap HP column (GE Healthcare, Munich, Germany) coupled to an Äkta-Purifier FPLC system (GE Healthcare) and washing buffer supplemented with 40 mM imidazole. ANAC42-IFP-His was enriched using Protino Ni-IDA 150 packed column (Macherey-Nagel, Düren, Germany), in the presence of 1 mM EDTA to inhibit metal proteases, and washing buffer without imidazole.

### Western blot and infrared analysis

Protein samples were separated in 12% SDS-polyacrylamide gels under denaturing conditions and analysed either (i) immunologically or by (ii) infrared scanning. (i) For immunological analysis SDS-PAGE-separated proteins were transferred onto Protran nitrocellulose membrane (Whatman, Kent, UK). The membrane was blocked for one hour in blocking buffer (5% non-fat dry milk in PBS containing 0.1% Tween-20), followed by incubation for 1 h with monoclonal mouse antibody (Santa Cruz Biotechnology, California, USA) directed against the 6xHis epitope. Membranes were washed three times for 10 min in wash buffer (PBS containing 0.1% Tween-20) and incubated for 1 h with IRDye800CW-conjugated goat anti-mouse secondary antibody (LI-COR, Bad Homburg, Germany). All incubations were performed at room temperature and antibodies were diluted 1:10,000 in blocking buffer. Signal intensities were analysed at 800 nm by using the Odyssey Infrared Imaging System (LI-COR). (ii) IFP-mediated infrared fluorescence was detected with the same system but scanning at 700 nm.

IFP was measured directly after transfer of IFP-His or ANAC42-IFP-His fusion protein expressing cells (2-100 μL) into the wells of black 96-well ELISA plates with clear flat bottom (Corning, New York, USA). If not stated otherwise, cell cultures were directly scanned in the wells of the ELISA plate; otherwise plates were centrifuged for 2 min at room temperature and 2500 rpm followed by scanning. In-gel detection was done after SDS-PAGE without demounting cast protein gels of the Mighty Small II system (Hoefer, Massachusetts, USA). Separated proteins were also visualized by Coomassie staining after infrared analysis.

### Deconvolution microscopy

For imaging of IFP in individual *Leishmania *cells fluorescence images were taken using a Zeiss Cell Observer HS/Axiovert 200 M deconvolution microscope (Carl Zeiss MicroImaging, Göttingen, Germany) equipped with a Cy5.5 filter set (665 ± 22.5 nm excitation and 725 ± 25 nm emission).

## Results and Discussion

### Expression of IFP in *L. tarentolae*

**Figure 1:**
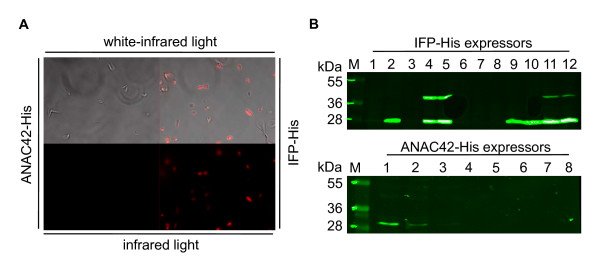
**Imaging of IFP in *Leishmania *cells and Western blot analysis**. A) Fluorescence images of *Leishmania *cells expressing IFP-His or ANAC42-His fusion protein were taken with a Cy5.5 filter set (665 ± 22.5 nm excitation and 725 ± 25 nm emission) using deconvolution microscopy. Illumination with white and infrared light simultaneously visualizes *Leishmania *cells and infrared signal, whereas infrared light detects infrared signal only. Infrared fluorescence signal is only detected in IFP-expressing cells, but not in cells expressing IFP-free negative control protein ANAC42-His. B) Several *Leishmania *cell lines (twelve IFP-His and eight ANAC42-His clones) were randomly selected and molecular weights of the expressed fusion proteins - 37 kDa for IFP-His and 33 kDa for ANAC42-His - were determined by SDS-PAGE analysis. Pre-stained markers were labeled on the membrane with a blue ball pen resulting in green-fluorescing dots upon excitation at 800 nm using the Odyssey Infrared Imaging System.

In order to investigate the potential usage of IFP as a reporter for protein expression in *L. tarentolae *we expressed it in the cytoplasm and analyzed the cells by deconvolution microscopy (Figure 1A). A combination of white light with infrared imaging was chosen for simultaneous visualization of motility and shape of *Leishmania *cells and detection of infrared fluorescence, whereas infrared excitation was chosen for the detection of the infrared signal only. We detected pink or red fluorescing parasite cells under white/infrared and infrared light, respectively, only when IFP was expressed. No background signal was observed when ANAC42, a transcription factor from the plant *Arabidopsis thaliana*, was expressed as a negative control, indicating the absence of infrared light-emitting components from the culture broth or untransformed *Leishmania *cells. IFP expressing *Leishmania *cells showed normal motility and shape, and growth rate was identical to non-transformed control cells and cells expressing ANAC42 (not shown), indicating that intracellular accumulation of the fluorescent protein caused low or no toxicity. Furthermore, infrared imaging showed that IFP is expressed throughout the cells. These data document, that IFP can be employed as a facile reporter for protein expression in *Leishmania*. Additionally, IFP may be used in other experimental procedures such as for instance infrared fluorescence-activated cell sorting to isolate *Leishmania *cells expressing IFP at high level.

Expression levels of IFP and ANAC42, both carrying a 6xHis-tag at their C-terminal end, and their respective molecular weights were verified by SDS-PAGE and Western blot analysis (Figure 1B). Following the recommended procedural pipeline for protein expression in *Leishmania *(documented in the manual to the LEXSYcon2 Expression Kit; Jena Bioscience), we analyzed individual clones for accumulation of the heterologous proteins and identified four IFP-6xHis (no. 4, 5, 11 and 12) and three ANAC42-6xHis (no. 1, 2 and 3) clones expressing fusion protein (Figure 1B).

### The role of hemin and biliverdin in IFP expressing *Leishmania *cells

**Figure 2:**
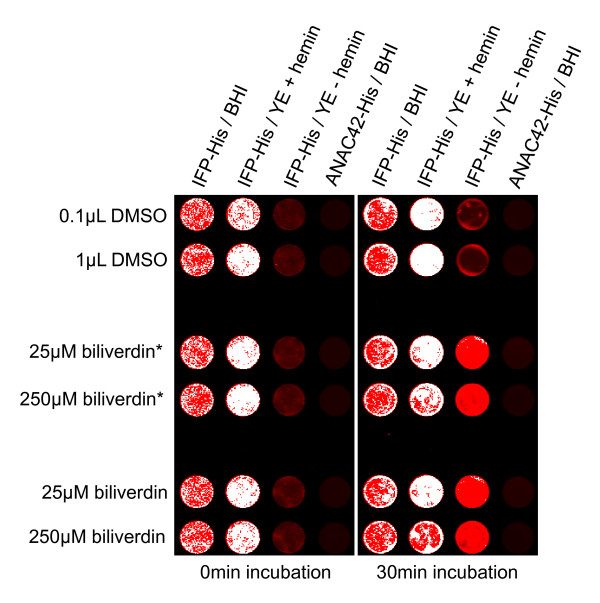
**Hemin is essential for growth of *Leishmania *and IFP fluorescence**. IFP-His expressing cell line was grown in BHI medium (BHI), YE medium supplemented with hemin (YE + hemin) or YE medium lacking hemin (YE - hemin). An ANAC42-His expressing cell line grown in BHI medium was used as a negative control. Hundred μL of the cultures were transferred to wells of a 96-well ELISA plate and supplemented with 25 μM and 250 μM of biliverdin (*; expensive) or biliverdin hydrochloride (lower price; stock solutions 25 mM in DMSO). DMSO alone was used as negative control. After addition of biliverdin or biliverdin hydrochloride, respectively, samples were incubated at 26°C. Samples were IR-scanned at 700 nm before and 30 min after addition of biliverdin or biliverdin hydrochloride using the Odyssey Infrared Imaging System. Moderate IFP fluorescence is seen in red, whereas intense IFP signal appears as white pixels in the image. Note the very low IFP fluorescence in IFP-His cultures lacking hemin (0 min incubation), and the complete absence of IFP signal in cells expressing negative control protein ANAC42-His.

Biliverdin, a green tetrapyrrolic pigment, is a product of heme catabolism. The biliverdin chromophor spontaneously and covalently incorporates into IFP resulting in infrared fluorescence upon excitation at 684 nm. Expression of IFP in *Leishmania *resulted in infrared fluorescence in the presence of hemin, which is essential for *Leishmania *growth, even when external biliverdin was omitted from the BHI culture broth (Figure 2), indicating that hemin can be catabolized into biliverdin by *Leishmania*. In addition we cultured IFP-expressing *Leishmania *cells in YE media with or without hemin. The *Leishmania *cultures were scanned for infrared fluorescence resulting in IR signal in hemin-containing media only. External addition of biliverdin (Toronto Research Chemicals) or biliverdin hydrochloride (Frontier Scientific) to hemin-lacking culture resulted in IR signal recovery after 30 min of incubation (Figure 2). Biliverdin used by Shu *et al*. was purchased from Toronto Research Chemicals [12]. However, these authors proposed that biliverdin hydrochloride might be used alternatively, however tests were not performed (as indicated on the Tsien lab web page). Here, in Figure 2, we show that biliverdin hydrochloride (<1 US-$/mg) can replace the more expensive biliverdin (~40 US-$/mg), keeping costs associated with IFP reporter expression low.

### IFP allows sensitive and rapid identification of well-expressing *Leishmania *clones

**Figure 3:**
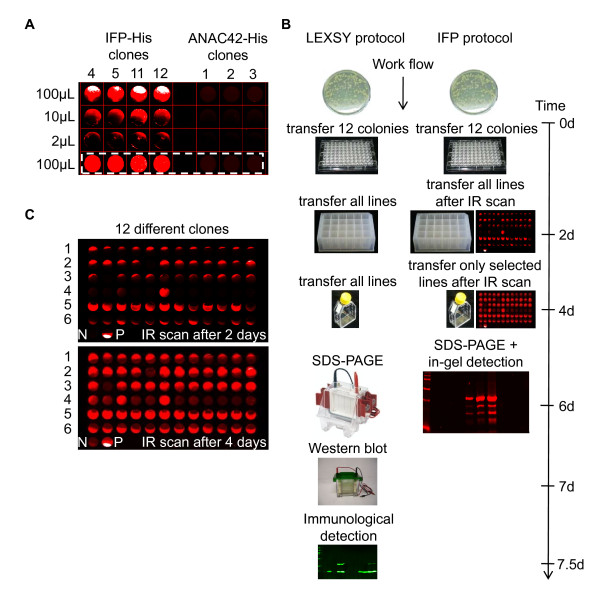
**Identification of well-expressing *Leishmania *strains**. A) Four IFP-His and three ANAC42-His fusion protein-expressing *Leishmania *cell lines (clones 4, 5, 11, 12 and 1, 2, 3, respectively) of different volumes (2 μL, 10 μL and 100 μL) were transferred into the wells of a 96-well ELISA plate and centrifuged for 2 min at 2,500 *g*, followed by pipeting again 100 μL of the respective cell lines into neighbouring wells indicated by a white-dashed box. IR scanning was performed using the Odyssey Infrared Imaging System. B) Schematic outline of the previously established LEXSY protocol and the IFP protocol described here. The flow-chart high-lights the differences of the two *Leishmania *protein expression protocols in terms of expenditures of time and effort. C) Twelve different IFP fusion protein (1, ARR1-IFP-His; 2, IFP-ARR1-His; 3, ANAC42-IFP-His; 4, IFP-ANAC42-His; 5, TPK1-IFP-His; 6, IFP-TPK1-His) expressing *Leishmania *cell lines were picked from the selective plate and transferred into the wells of a 96-well microtiter plate filled with 150 μL BHI medium, followed by two days of incubation and IR scan (upper panel). The complete volumes of all cell lines were transferred into the wells of a 24-well deep-well plate containing 1 mL BHI medium, followed by incubation for two days. 100 μL-samples of each cell line were IR-scanned in the wells of a 96-well microtiter plate (lower panel). IFP-His expressing cell line was used as positive control (P) and ANAC42-His expressing cell line as negative control (N).

In order to demonstrate the sensitivity of IFP as an *in vivo *detector for protein synthesis different IFP expressing cell lines were grown in 25 cm^2 ^tissue culture flasks in a culture volume of 10 mL, as recommended by the manufacturer. Subsequently 2 μL, 10 μL and 100 μL, respectively, of the cell culture were transferred into individual wells of a 96-well ELISA plate with a flat and clear bottom, and centrifuged. After centrifugation a second transfer of 100 μL culture volume into neighboring wells was carried out. Centrifuged and non-centrifuged IFP expressing *Leishmania *cell cultures were scanned directly in the ELISA plate. Cell lines expressing ANAC42 protein without a fusion to IFP were used as negative control. As can be seen in Figure 3A, culture volumes of as little as 2 μL are sufficient for straightforward detection of IFP expressing *Leishmania*; furthermore centrifugation to collect cells in a pellet is not required for *in vivo *IFP detection (dashed white box in Figure 3A). Thus, IFP fusion proteins are rapidly detected by IR measurement allowing pre-selection of well-expressing *Leishmania *cell lines at an early stage of the expression pipeline. In Figure 3B we compare the *Leishmania *protein expression work-flow (LEXSY protocol) recommended by the manufacturer, which uses 6xHis as reporter, with the work-flow reported here that employs IFP as marker (IFP protocol). According to the IFP protocol well-expressing *Leishmania *lines are visualized by IR scan in a 96-well microtiter plate already two days after transfer and incubation of individual clones in a culture volume of 150 μL. The complete culture volumes of each clone are then transferred into the wells of a 24-well deep-well plate (containing 1 mL culture broth each), followed by two additional days of incubation. IR scan in a 96-well microtiter plate, using 100 μL of each clone, enables a pre-selection of cell lines expressing IFP at moderate to high level. Subsequently, 1 mL culture of well-expressing clones is transferred into 10 mL culture broth in 25 cm^2 ^tissue culture flasks. After two further days of incubation, at day six, IFP or IFP fusion proteins are detected in-gel after separation by SDS-PAGE (IFP protocol in Figure 3B). In contrast, in the LEXSY protocol all cultures are scaled-up for the detection of 6xHis fusion proteins by Western blot analysis after seven to eight days in total. Thus, time (1 1/2 days) and in particular experimental effort required for the detection of protein expression in *Leishmania *can be reduced considerably when using IFP as reporter (Figure 3). The new protocol was approved by expressing different proteins fused to IFP at their N- and C-terminal ends, respectively, including ARR1, a response regulator in the plant cytokinin signaling pathway, ANAC42, a transcription factor, and the N-terminal GRF (General Regulating Factor)-interacting domain of TPK1 (Figure 3C). Ten μL each of 12 randomly selected cell lines of unknown cell densities were centrifuged and analyzed for IR fluorescence at two different stages of the newly established protein expression work-flow, namely two days after incubation in the 96-well microtiter plate (Figure 3C, upper panel) and two days after incubation in the 24-well deep-well plate (Figure 3C, lower panel). Taken together, the utilization of IFP as a reporter for expression of fusion proteins in *Leishmania *reduces time, costs and efforts and thus makes it an attractive reporter protein especially for high-throughput analyses in genomics research.

### Online detection of IFP for the analysis of protein expression and purification parameters

**Figure 4:**
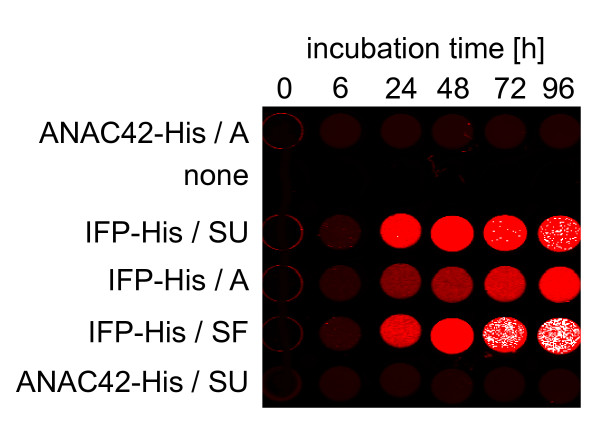
**Online detection of IFP in *L. tarentolae***. IFP-His expressing *Leishmania *cells were grown in 25 cm^2^-tissue culture flasks in 10 mL of BHI medium in static upright (SU), static flat (SF) and dynamic (60 rpm) flat (A) position. ANAC42-His expressing cells were used as negative control. After 0, 6, 24, 48, 72 and 96 h of incubation 100 μL-samples were IR-scanned in the wells of a 96-well microtiter plate.

**Figure 5:**
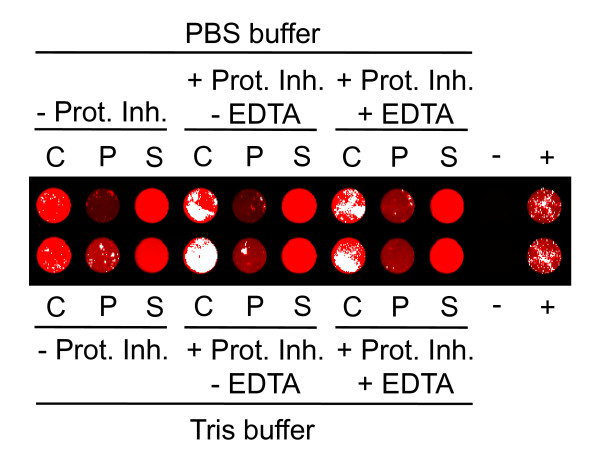
**Stability and solubility analysis of IFP expressed in *L. tarentolae***. IFP-His expressing *Leishmania *cells were sonicated in Tris and PBS buffer containing a protease inhibitor cocktail supplemented with EDTA (+Prot. Inh. + EDTA), an EDTA-free protease inhibitor cocktail (+Prot. Inh. - EDTA) or no protease inhibitors (- Prot. Inh.), and ultracentrifuged. Crude extracts (C) before ultracentrifugation, as well as pellets (P) and soluble supernatants (S) after ultracentrifugation were IR-scanned in the wells of a 96-well microtiter plate. (-) 100 μL ANAC42-His expressing *Leishmania *cells, negative control for IR signal; (+) 100 μL IFP-His expressing cells, positive control for IR signal.

**Figure 6:**
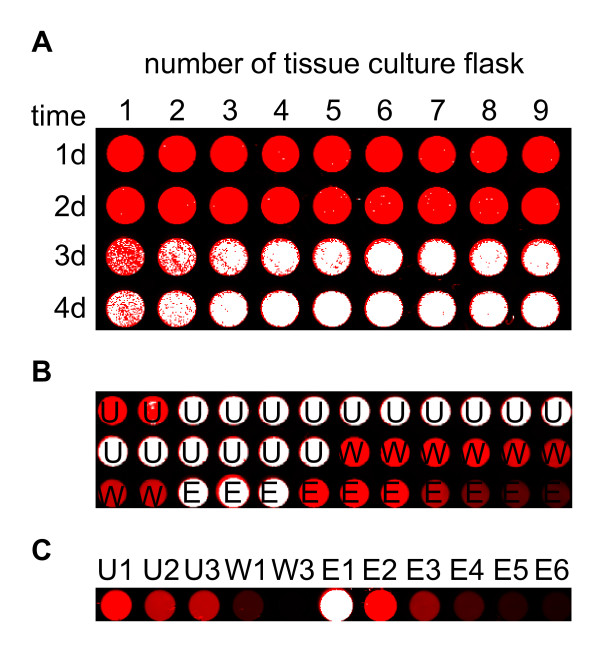
**Scale-up and purification of IFP fusion proteins expressed in *L. tarentolae***. A) Culture volume of IFP-His expressing *Leishmania *cells was scaled-up in nine 150 cm^2^-tissue culture flasks (1-9), followed by incubation under static flat condition for four days (1d-4d). Samples for IR analysis were taken every 24 h. B) Scaled-up culture volumes were pooled and IFP-His protein was purified by affinity chromatography using a HisTrap HP column coupled to the Äkta-Purifier FPLC system. Aliquots of the 1-mL unbound (U), wash (W) and elution (E) fractions were IR-scanned in the wells of a 96-well microtiter plate. C) Expression of ANAC42-IFP-His fusion protein was scaled-up and the protein was enriched manually on a Protino Ni-IDA 150 column by circulating the protein-containing sample three times. Aliquots of the fractions unbound after each circulation (U1, 2, 3), washing (W1, 3) and elution (E1 - E6) were IR-scanned in the wells of a 96-well microtiter plate. Red signal in the image indicates moderate and white signal high IFP concentration.

Production of large quantities of proteins with high yield requires optimal expression parameters. Once expression is optimized under laboratory conditions, the process can be scaled-up to the desired bioreactor volume followed by purification of the protein of interest. Besides optimizing gene sequences and choosing the right expression system, optimal expression yields can be achieved by changing culture and bioreactor parameters, e.g. media ingredients, pH value or O_2 _concentration in the culture broth. To demonstrate that IFP can be used as an online sensor allowing researchers to continuously and quantitatively monitor expression of IFP in real time, a 1:10 diluted IFP-expressing *Leishmania *cell culture was grown in 25 cm^2 ^tissue culture flasks in a volume of 10 mL under different mechanical conditions (static upright, static flat and dynamic flat). Changes in IR signal intensities due to changes of IFP quantities were measured at different time points by transferring and scanning 100 μL of the cell cultures directly into the wells of a 96-well ELISA plate without centrifugation (Figure 4). After 24 h of incubation the first IFP signal among all three incubation conditions was detected and a further increase of the signal was observed after two, three and four days, respectively. However, dynamic flat incubation resulted in only a slow increase of the IFP signal, while signal intensity increased rapidly when cells were incubated under static upright condition, and even faster when grown under static flat condition. In the latter case, high level of IFP was observed after 72 h and 96 h, visible as white pixels in the image (Figure 4). These results indicate that IFP expression under dynamic incubation condition can inhibit the production of IFP due to either excessive oxygen transfer into the culture broth or increased shear forces acting on the *Leishmania *cells due to movement of the culture flasks. Next we tested the stability and solubility of IFP in two different standard buffers used for the purification of His-tagged fusion proteins, in the absence and presence of different protease inhibitors. Figure 5 demonstrates that IFP alone is stable in Tris and PBS buffer in the absence of protease inhibitors and accumulates in the soluble supernatant after ultracentrifugation, which is a prerequisite for purification of proteins under native conditions. Cells were disrupted in the described buffers by ultrasound treatment indicating that sonication does not affect the infrared signal of IFP. IFP production was scaled-up by growing cells in parallel in nine 150 cm^2 ^tissue culture flasks in a culture volume of 60 mL. The flasks were incubated static flat for four days, and the development of IR signal in each flask was monitored every 24 h (Figure 6A). All culture broths were pooled and centrifuged on the fourth day. The resulting cell pellet was disrupted in 25 mL Tris buffer with an EDTA-free protease inhibitor cocktail and IFP-His fusion protein was purified by Äkta-FPLC using a 1-mL His-Trap column. Unbound flow-through, as well as flow-through of washing and elution steps were collected (1-mL fractions). Hundred μl of each fraction were analyzed by IR scan allowing the detection of bound and partially unbound IFP-His fusion protein (Figure 6B). The amount of unbound protein can be reduced by collecting and recirculating the unbound flow through several times through the column. This was demonstrated by using the ANAC42-IFP-His fusion protein and recirculating it three times manually through a gravity flow-based 6xHis-affinity column (Figure 6C).

### In-gel detection of IFP and IFP fusion proteins

**Figure 7:**
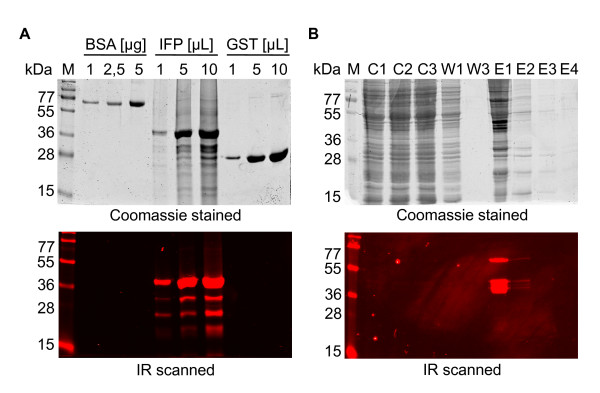
**In-gel detection of IFP fusion proteins expressed in *L. tarentolae***. A) Purified IFP-His fusion protein, BSA and purified GST were separated by SDS-PAGE. In-gel detection was performed directly in the cast gel (lower panel), followed by Coomassie staining for visualization of all protein bands (upper panel). Partial protein degradation was observed in the IR scan, most likely because the metal protease inhibitor EDTA was omitted (to preserve the HisTrap HP columns used). B) Abundance of ANAC42-IFP-His protein during affinity chromatography was tested by analyzing aliquots of the following fractions: C1, 2, 3, unbound protein after each circulation; W1, 3, protein in wash solution; E1 - E4, protein after elution. Proteins were separated by SDS-PAGE and scanned in-gel (lower panel) and subsequently subjected to Coomassie staining (upper panel). M, molecular size marker.

Expression and online in-cell detection as well as purification and enrichment of the IFP-His and ANAC42-IFP-His fusion proteins from *Leishmania *cells were shown above. Next we separated IFP fusion proteins by gel electrophoresis under denaturing conditions (SDS-PAGE) and found that IR scanning detects IFP fusion proteins directly in the casted protein gel (Figure 7). In Figure 7A different amounts of bovine serum albumin (BSA), purified IFP-His fusion protein and purified GST protein were separated and scanned for IR signals (lower panel), followed by Coomassie staining (upper panel) of the protein gel. The IR image demonstrates that IFP-His fusion protein is specifically detected in the gel when excited at 700 nm. Although an excess of BSA or GST was applied to the gel they were not detected by IR excitation, indicating very low background signal. Similar results were obtained for ANAC42-IFP-His fusion protein (Figure 7B). The comparison of the results obtained by IR scanning and Coomassie staining of the protein gel in Figure 7A clearly shows that the IR detection limit of IFP is less than 1 μg. However, expression level of ANAC42-IFP-His fusion protein was considerably lower than that of IFP-His. We observed that in general, at a population density of 9 × 10^8 ^cells/mL, 50 ng to 2 μg IFP fusion protein can be produced per mL of *Leishmania *cell culture, depending on the protein and the N- or C-terminal positioning of IFP (data not shown).

## Conclusions

We demonstrated that IFP can be used as a facile reporter protein in *L. tarentolae*. Our study shows that IFP can be expressed in *Leishmania *cells to a level that allows easy detection by the Odyssey Infrared Imaging System, without compromising cell growth. Using IFP as biosensor expression of IFP and IFP fusion proteins in *Leishmania *is easily and rapidly detected in-cell, without cell disruption in microtiter plates. A few microliters of expression cultures are sufficient for in-cell detection and pre-selection of well-expressing *Leishmania *clones at an early stage of the protein production pipeline. Our IFP-based work-flow significantly reduces not only hands-on-time and work load for the individual researcher but also preserves precious cell culture, particularly when culture volumes and cell densities are low initially, when an expression protocol is established for a new protein, or when proteins are expressed weakly. Importantly, addition of biliverdin to the culture medium is not required to achieve infrared fluorescence of IFP-expressing *Leishmania *cells. Instead, hemin, a component essential for *Leishmania *growth, is sufficient for IFP chromophore formation, probably because of its intracellular enzymatic conversion to biliverdin.

As a further incentive, IFP fusion proteins retain infrared fluorescence after electrophoresis in denaturing SDS-polyacrylamide gels, allowing their direct in-gel detection by infrared imaging without disassembling cast gels. Thus, parameters for scaling up protein production and streamlining purification routes can be easily optimized when employing IFP as reporter. We envisage that expression of IFP-labelled proteins in *Leishmania *will assist the genomics-driven analysis or proteins from plants and other model organisms.

## Competing interests

The authors declare that they have no competing interests.

## Authors' contributions

Both authors, HD and BM-R, devised the research and experimental procedures. HD performed all experiments. BM-R supervised the research. Both authors wrote the manuscript.
